# Use of S-ICD tunneling tool for percutaneous epicardial access during ventricular tachycardia ablation

**DOI:** 10.1007/s10840-025-02113-9

**Published:** 2025-08-11

**Authors:** Jackson J. Liang, Nithi Tokavanich, Frank Bogun

**Affiliations:** Section of Electrophysiology, Division of Cardiology, Department of Internal Medicine, University of Michigan, 1500 E Medical Center Dr, Ann Arbor, MI 48109 USA

A 61-year-old man with nonischemic cardiomyopathy and ventricular tachycardia (VT) who had undergone endocardial ablation 6 months prior was referred for repeat VT ablation due to recurrent VT and implantable cardioverter defibrillator shocks. Cardiac MRI showed transmural basal lateral delayed enhancement.

Programmed stimulation at the beginning of the procedure induced multiple VT morphologies with suspected epicardial exits. As such, the decision was made to obtain epicardial access before systemic heparinization. Under conscious sedation (fentanyl and midazolam), a subxiphoid incision was created 1 cm beneath the xiphoid process. Biplane fluoroscopy (Fig. 1A and B) revealed gastric air bubble anteriorly along the anticipated needle course. To avoid risk of gastric puncture, a subcutaneous implantable cardioverter defibrillator (S-ICD) sternal lead tunneling device with pre-loaded sheath (Boston Scientific, Marlborough, MA) was used to bluntly tunnel a tract from the subxiphoid incision beyond the stomach and diaphragm towards the anterior aspect of the right ventricle. After advancement beyond the diaphragm was confirmed with biplane fluoroscopy, the tunneling tool was removed and the sheath was left in place. A bevel-tipped Tuohy epidural needle (18 gauge, 8 inch length) was advanced through the sheath (Fig. 1B) and the pericardium was punctured. An 0.032″ J-tipped wire was advanced into the pericardial space and confirmed to cross all four cardiac chambers (Fig. 1C and D) before a steerable sheath was inserted to facilitate epicardial mapping and ablation. Aspiration after epicardial access confirmed absence of bleeding. Systemic heparin was administered and successful epicardial and endocardial ablation was performed rendering him noninducible for VT with programmed stimulation at the end of the procedure. A pericardial drain was left in place prophylactically which was removed 24 h later without complications.

**Fig. 1:**
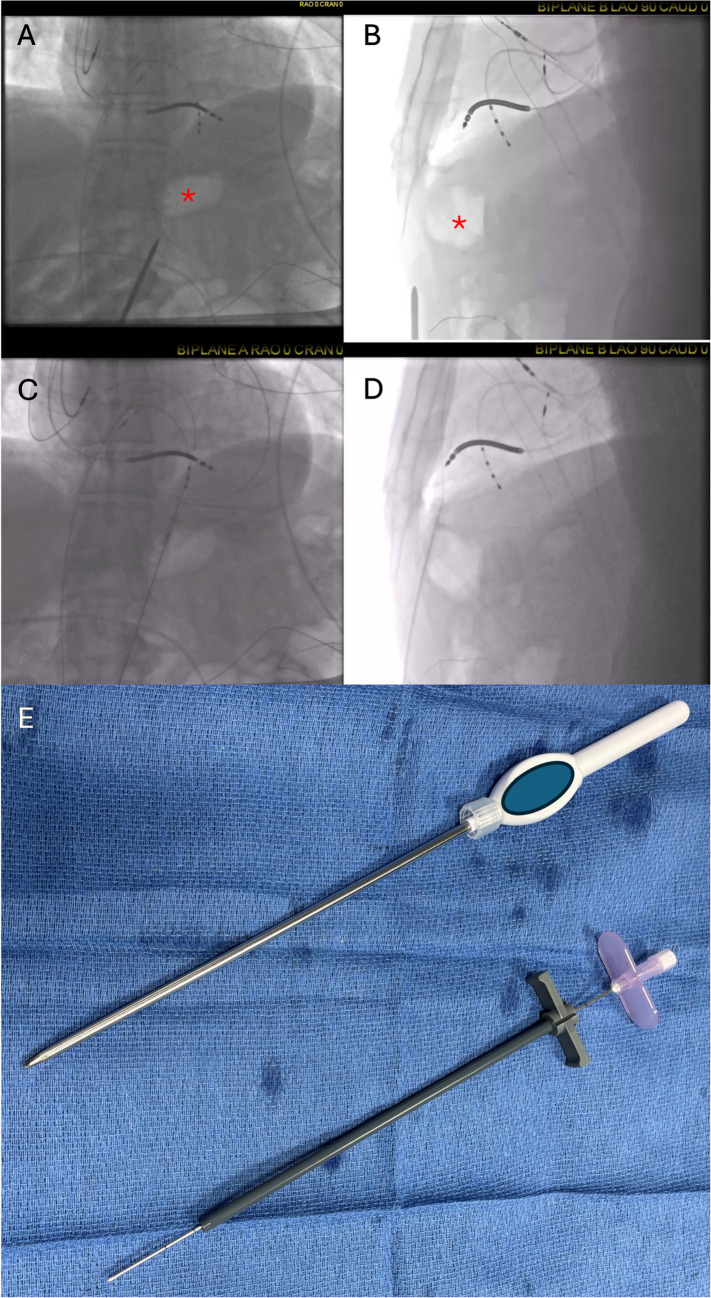
Panels **A** (AP) and **B **(LAO 90) show gastric air bubble (star) anteriorly along course of needle. Panels **C** (AP) and **D** (LAO 90) show wire in pericardial space after advancement of needle through sheath which had been advanced with tunneling tool beyond gastric air bubble. Panel **E** shows S-ICD tunneling tool and insertion of Tuohy needle (18 gauge, 8 inch length) through sheath

While strategies such as needle-in-needle access, CO2 insufflation (via intentional coronary vein or right atrial appendage perforation) may decrease risk of significant bleeding due to right ventricular puncture, they do not reduce risk of thoracic arterial injury and damage to subdiaphragmatic structures caused by needle advancement en route to the heart. Damage to subdiaphragmatic structures including the liver, bowel, and stomach can occur during percutaneous epicardial access, especially when posterior approach is attempted. Meanwhile, anterior approach can increase risk of arterial injury (internal thoracic, superior epigastric, and musculophrenic arteries). Use of the S-ICD tunneling device allows for blunt sheath advancement beyond the diaphragm through which a needle can be advanced for pericardial access (Fig. 1E). This simple and cost-effective technique may improve safety of epicardial access, minimizing risk of both arterial injury and damage to subdiaphragmatic structures, and can be readily used in conjunction with other strategies focused on minimizing bleeding due to RV puncture (CO2 insufflation, needle-in-needle, etc.).

## Supplementary Information

Below is the link to the electronic supplementary material.

Supplementary file1 (MP4 3696 KB)

Supplementary file2 (MP4 3198 KB)

